# Characterization of macrophage - cancer cell crosstalk in estrogen receptor positive and triple-negative breast cancer

**DOI:** 10.1038/srep09188

**Published:** 2015-03-17

**Authors:** Maija Hollmén, Filip Roudnicky, Sinem Karaman, Michael Detmar

**Affiliations:** 1 Institute of Pharmaceutical Sciences, Swiss Federal Institute of Technology, ETH Zürich, 8093 Zurich, Switzerland

## Abstract

Tumor heterogeneity may broadly influence the activation of tumor-associated macrophages. We aimed to dissect how breast cancer cells of different molecular characteristics contribute to macrophage phenotype and function. Therefore, we performed whole transcriptome sequencing of human monocytes that were co-cultured with estrogen receptor positive (ER^+^) or triple-negative (TNBC) breast cancer cell lines and studied the biological responses related to the differential gene activation in both monocytes and cancer cells by pathway analysis. ER^+^ and TNBC cancer cell lines induced distinctly different macrophage phenotypes with different biological functions, cytokine and chemokine secretion, and morphology. Conversely, ER^+^ and TNBC breast cancer cell lines were distinctly influenced by the presence of macrophages. ER^+^ cells demonstrated up-regulation of an acute phase inflammatory response, IL-17 signaling and antigen presentation pathway, whereas thioredoxin and vitamin D_3_ receptor pathways were down-regulated in the respective macrophages. The TNBC educated macrophages down-regulated citrulline metabolism and differentiated into M2-like macrophages with increased MMR protein expression and CCL2 secretion. These data demonstrate how different cancer cells educate the host cells to support tumor growth and might explain why high infiltration of macrophages in TNBC tumors associates with poor prognosis.

Tumor-associated macrophages (TAMs) are generally thought to display immunosuppressive properties with low antigen presenting capability, low cytotoxic functions and high tissue remodeling activity^1^. These functions either directly or indirectly promote tumor growth by increasing tumor cell survival, proliferation, invasiveness and motility. The immunosuppressive TAMs are activated by Th2 cytokines (interleukin (IL) -4 and IL-13) as well as other immunosuppressive molecules such as IL-10, glucocorticoids and vitamin D_3_ and they have functions of alternatively activated M2-type macrophages^2^. Interestingly though, recent findings suggest that not all TAMs exhibit similar properties and depending on the location, tumor type and stromal interactions, some TAMs, even within the same tumor, are Th1 skewed, classically activated M1-type macrophages^3^. In contrast to M2-type macrophages, M1-type macrophages are pro-inflammatory and promote anti-tumor immune responses to facilitate tumor destruction^4^. As such, a high tumor infiltration with TAMs has been associated with favorable prognosis in gastric cancer^5^, some colorectal carcinomas^5^,^6^ and melanoma^7^.

On the other hand, extensive infiltration of TAMs in bladder^8^, prostate^9^, endometrial^10^ and kidney^11^ cancer correlates with poor prognosis and increased tumor angiogenesis. Clinical data from human invasive breast cancer samples show that the abundance of TAMs correlates with high tumor grade, low hormone receptor status and reduced relapse-free and overall-survival^12^,^13^. Moreover, high expression of CCL2 (monocyte chemotactic protein 1, MCP-1) on both macrophages and tumor cells in primary breast cancer has been shown to correlate significantly with intensive TAM accumulation, high expression of vascular endothelial growth factor (VEGF) and early relapse^14^. These observations are possibly linked to the ability of CCL2 to recruit inflammatory monocytes that facilitate breast tumor metastasis^15^, and to inhibit the generation of tumor-reactive T cells by enhanced IL-4 production on T-cells^16^. All together, TAMs seem to have an unfavorable role in breast cancer. However, breast cancer is considered to be a highly heterogeneous disease from a molecular point of view, and many secreted factors might differentially contribute to macrophage phenotype in different breast cancer types. Therefore, our aim was to understand the host-tumor interactions in a simplified setting by comparing macrophage activation under the influence of two immunopathologically classified breast cancer types, namely estrogen receptor positive (ER^+^) and triple-negative (TNBC) breast cancer. ER^+^ tumors comprise ~75% of all human breast cancers. They are less aggressive and respond well to anti-hormonal therapy. TNBC represent 10–20% of all breast cancers and is associated with African-American race, younger age, *BRCA1* mutations and poorer prognosis^17^. TNBC is a very aggressive cancer with relatively poor prognosis due to its heterogeneity, resistance to current treatments and lack of efficient targeted therapies^18^.

Here we show that human monocytes that are activated by the TNBC tumor cell line MDA-MB-231 have a dramatically different phenotype compared to those activated by the ER^+^ tumor cell line T47D. These changes, in turn, resulted in opposite effects of these cells on the proliferation of cancer cells. Next generation sequencing revealed gene expression signatures, comprising several metabolic pathways governing M1 vs. M2 macrophage activation and immune activation pathways, that reflected the biological differences seen between ER^+^ and TNBC.

## Results

### Human monocytes selectively differentiate into M2-like macrophages when co-cultured with a TNBC cell line

**Figure 1:**
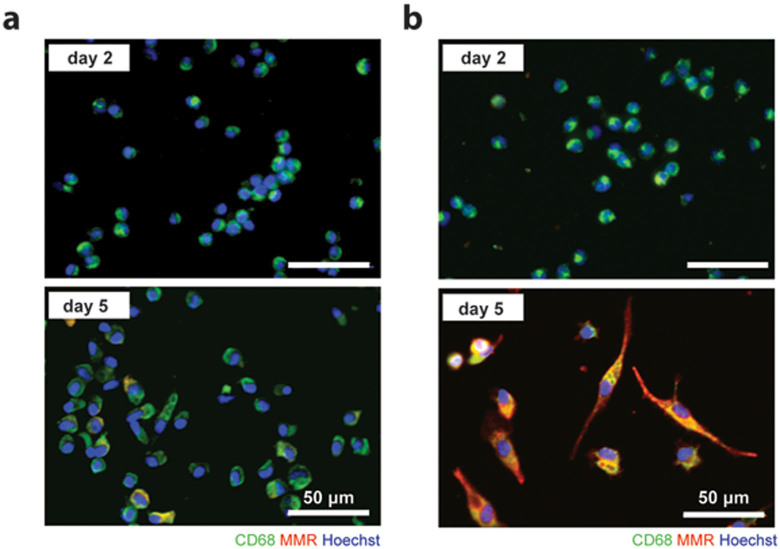
MDA-MB-231 activated macrophages obtain an M2-like polarization. Monocyte differentiation into macrophages under transwell (tw) co-culture with (a) T47D (ER^+^) or (b) MDA-MB-231 (TNBC) cells was visualized on day 2 and day 5 by immunofluorescence analysis, using the common macrophage marker CD68 (green) and a marker for M2-like macrophages, macrophage mannose receptor (MMR; red).

To characterize the differences in macrophage activation under the influence of either ER^+^ or TNBC breast cancer cells, we cultured freshly isolated human peripheral monocytes with two breast cancer cell lines (T47D, ER^+^ and MDA-MB-231, TNBC) in an in vitro transwell co-culture assay. The transwell setting allowed us to investigate the effect of soluble mediators on macrophage activation since direct cell contact of these cells was inhibited by a polyethylene terephthalate (PET) membrane (pore size 0.4 *μ*m). The macrophages under T47D co-culture appeared morphologically mainly round, and some cells slightly up-regulated the macrophage mannose receptor (MMR), a commonly used marker to define M2 macrophages (Fig. 1a). In contrast, a dramatic change in the morphology of macrophages was observed under MDA-MB-231 co-culture, which was accompanied by a strong up-regulation of MMR on day five (Fig. 1b).

### Macrophage-cancer cell crosstalk induces major gene expression changes in both cell types

**Figure 2:**
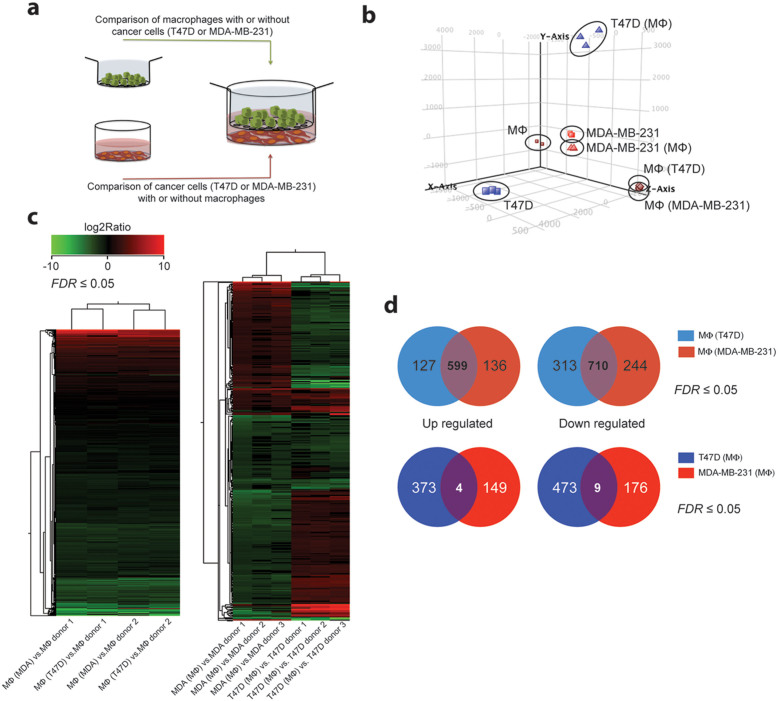
Gene expression changes under T47D and MDA-MB-231 cancer cell - macrophage co-culture conditions. (a) Experimental setup of whole transcriptome analysis for RNA-seq. (b) Principal component analysis (PCA) of whole transcriptome of individually cultured macrophages (brown squares, n = 2) and of T47D (brown circles, n = 2) or MDA-MB-231 (brown diamonds, n = 2) co-cultured macrophages; individually cultured T47D cells (blue squares, n = 3) and co-cultured T47D cells (blue triangles, n = 3); individually cultured MDA-MB-231 cells (red squares, n = 3) and co-cultured MDA-MB-231 cells (red triangles, n = 3) shows gene expression variance across the samples. 76.8% of the variance of the data is represented in the scatterplot and divided into three components: PC1 explains 52.84% of the variance data (x-axis), PC2 explains 34.47% of the variance of the data (y-axis) while PC3 explains 12.68% of the variance of the data (z-axis). (c) Heat maps of gene expression changes in both co-cultured macrophages and cancer cells compared to single culture conditions, respectively. Unsupervised hierarchical clustering was performed on the log_2_ ratio of significantly (*FDR* < 0.05) up- (red) and down- (green) regulated genes, using Euclidean distance matrix and average linking. (d) Venn diagrams summarizing differentially expressed genes after T47D (n = 2, light blue) or MDA-MB-231 (n = 2, light red) co-culture in macrophages (upper circles) and in T47D (n = 3, blue) and MDA-MB-231 (n = 3, red) cells after co-culture with macrophages (lower circles). Up-regulated genes: log_2_Ratio ≥ 1.5, *FDR* ≤ 0.05; down-regulated genes log_2_Ratio ≤ −1.5, *FDR* ≤ 0.05.

To investigate the distinct monocyte differentiation observed in co-culture with different tumor cells lines in more detail, we sequenced the whole transcriptome of both macrophages and cancer cells on day five of co-culture and compared their gene expression profiles to individually cultured cells (Fig. 2a). By using principal component analysis (PCA) to analyze for gene variances across our data set, we observed that both cancer cells induced a common change in gene expression of co-cultured macrophages compared to single cultured macrophages. These changes included the up-regulation of the known M2 markers *MRC1*, *STAB1*, *TGM2* and *CD163*; however, the fold induction of these genes was higher in the MDA-MB-231 educated macrophages compared to the T47D educated macrophages. We also noted a high variance of gene expression between T47D cells cultured alone or in co-culture with macrophages (Fig. 2b). Heatmaps of unsupervised hierarchical clustering, using gene sets with a false discovery rate (FDR) adjusted *P*-value of ≤0.05, demonstrated clustering of co-culture activated genes in macrophages by the monocyte donor rather than the type of co-cultured cancer cells (Fig. 2c, left heatmap). In contrast, the differentially expressed genes (DEGs) in the co-cultured cancer cells were not influenced by different monocyte donors but demonstrated cell type-specific gene expression changes with only minor overlap (Fig. 2c, right heatmap).

To delineate the gene expression differences in co-cultured macrophages defined by cancer cell type, we selected genes with a log_2_ ratio ≥ 1.5 that had changed in both donors (n = 2) cultured either with T47D cells (up-regulated genes n = 127 and down-regulated genes n = 313) or MDA-MB-231 cells (up-regulated genes n = 136 and down-regulated genes n = 214 (Fig. 2d, upper Venn diagrams). A similar cut off was performed for the gene expression changes occurring in either T47D cells co-cultured with three donor monocytes (up-regulated genes n = 373 and down-regulated genes n = 473) or MDA-MB-231 cells (up-regulated genes n = 149 and down-regulated genes n = 176) (Fig. 2d, lower Venn diagrams).

### T47D versus MDA-MB-231 activated macrophages activate different biological pathways that modulate pro- or anti-inflammatory responses

**Figure 3:**
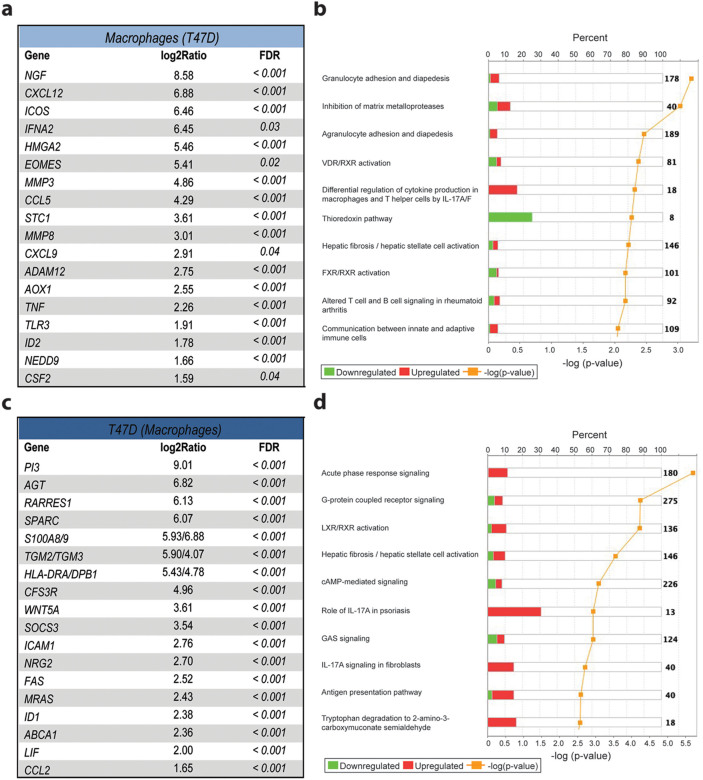
Co-culture induced gene expression changes and pathway analysis of biological responses in macrophages and T47D cells. (a) List of the most up-regulated inflammatory and cancer related genes in macrophages co-cultured with T47D cells and (b) ten most significantly altered pathways in co-cultured macrophages, analyzed using the differentially expressed gene sets by Ingenuity Pathway Analysis software. (c) List of genes up-regulated in co-cultured T47D cells and (d) ten most significantly altered pathways. The percent label indicates how many genes in a specific pathway were changed in the data set. Green represents down-regulated genes and red up-regulated genes in a specific pathway. The yellow label shows the significant change (−log (p-value)) of each pathway, generated using Fisher exact test.

In T47D-activated macrophages, we detected up-regulation of genes that mediate pro-inflammatory signals and leukocyte recruitment, such as *ICOS, IFNA2, TNF, TLR3*, *CXCL12, CCL5, CXCL9*, and *CSF2* (Fig. 3a). To address how the DEGs in these cells corresponded to changes in biological functions, we analyzed all up- and down-regulated DEGs using the Ingenuity Pathway Analysis software. The top ten most significantly altered pathways in T47D activated macrophages included agranulocyte/granulocyte adhesion and diapedesis, inhibition of matrix metalloproteases and IL-17 regulation (Fig. 3b). The thioredoxin and VDR/RXR activation pathways were significantly down-regulated, proposing M1-type activation of these cells^19^.

The T47D cells under co-culture responded to macrophage signals by up-regulating *S100A8, S100A9, TGM2, TGM3, CFSR3, WNT5A, ICAM, MRAS, ID1* and *CCL2*, which are all known mediators of tumor progression (Fig. 3c). However, breast cancer associated tumor suppressor genes such as *RARRES1, SPARC, SOCS3*, and *LIF* were also up-regulated in T47D cells. We also found an enhanced up-regulation of MHC I and II molecules (*HLA-DRA* and *HLA-DPBI* among others) and *FAS* (cell surface death receptor) on T47D cells after macrophage co-culture. These gene expression changes corresponded to the activation of acute phase response signaling, IL-17A signaling and antigen presentation pathways on T47D cells (Fig. 3d).

**Figure 4:**
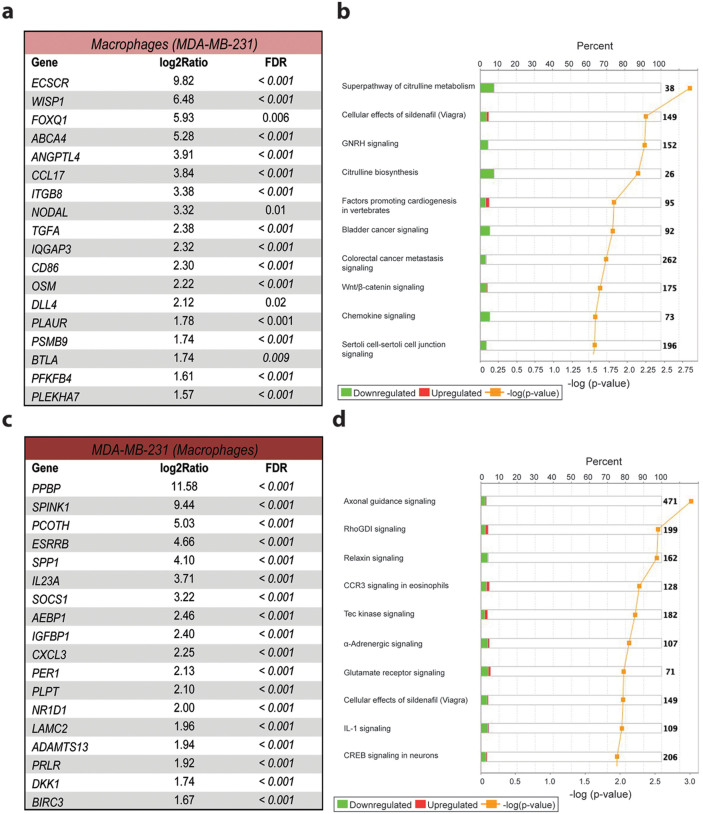
Co-culture induced gene expression changes and pathway analysis of biological responses in macrophages and MDA-MB-231 cells. (a) List of the most up-regulated inflammatory and cancer related genes in macrophages co-cultured with MDA-MB-231 cells and (b) ten most significantly altered pathways, analyzed using the differentially expressed gene sets by Ingenuity Pathway Analysis software. (c) List of genes up-regulated in co-cultured MDA-MB-231 cells and (d) ten most significantly altered pathways. The percent label indicates how many genes in a specific pathway were changed in the data set. Green represents down-regulated genes and red up-regulated genes in a specific pathway. The yellow label shows the significant change (−log (p-value)) of each pathway, generated using Fisher exact test.

In MDA-MB-231 co-cultured macrophages, genes involved in endothelial cell behavior (*ECSCR, ANGPTL4*, and *ITGB4*) and maintenance of stem cell renewal (*WISP1, NODAL, and DLL4*) were up-regulated (Fig. 4a). There was also an up-regulation of the epidermal growth factor receptor ligand transforming growth factor (TGF) α and oncostatin M, which is a mediator of epithelial to mesenchymal transition (EMT)^20^ and is expressed by M2-type macrophages^21^.

After co-culture with macrophages, MDA-MB-231 cells up-regulated genes involved in stem cell maintenance (*PPBP* and *CXCL3*)^22^, invasion (*SPINK1* and *LAMC2*), metastasis (*IGFBP1*), EMT (*SPP1*)^23^ and immunosuppression (*IL-23A*)^24^ (Fig. 4c). A general observation in the MDA-MB-231 macrophage co-culture was down-regulation of pathways such as citrulline metabolism and synthesis on macrophages and cellular effects of sildenafil on both macrophages (Fig. 4b) and cancer cells (Fig. 4d). Sildenafil (Viagra) is an inhibitor of phosphodiesterase-5 (PDE5) and prevents the degradation of cyclic guanosine monophosphate (cGMP). cGMP is produced from nitric oxide (NO) and therefore most likely affected by the down-regulation of the citrulline pathway.

### T47D co-culture induced secretion of pro-inflammatory factors CXCL10, IL-R2A and IL-3

**Figure 5:**
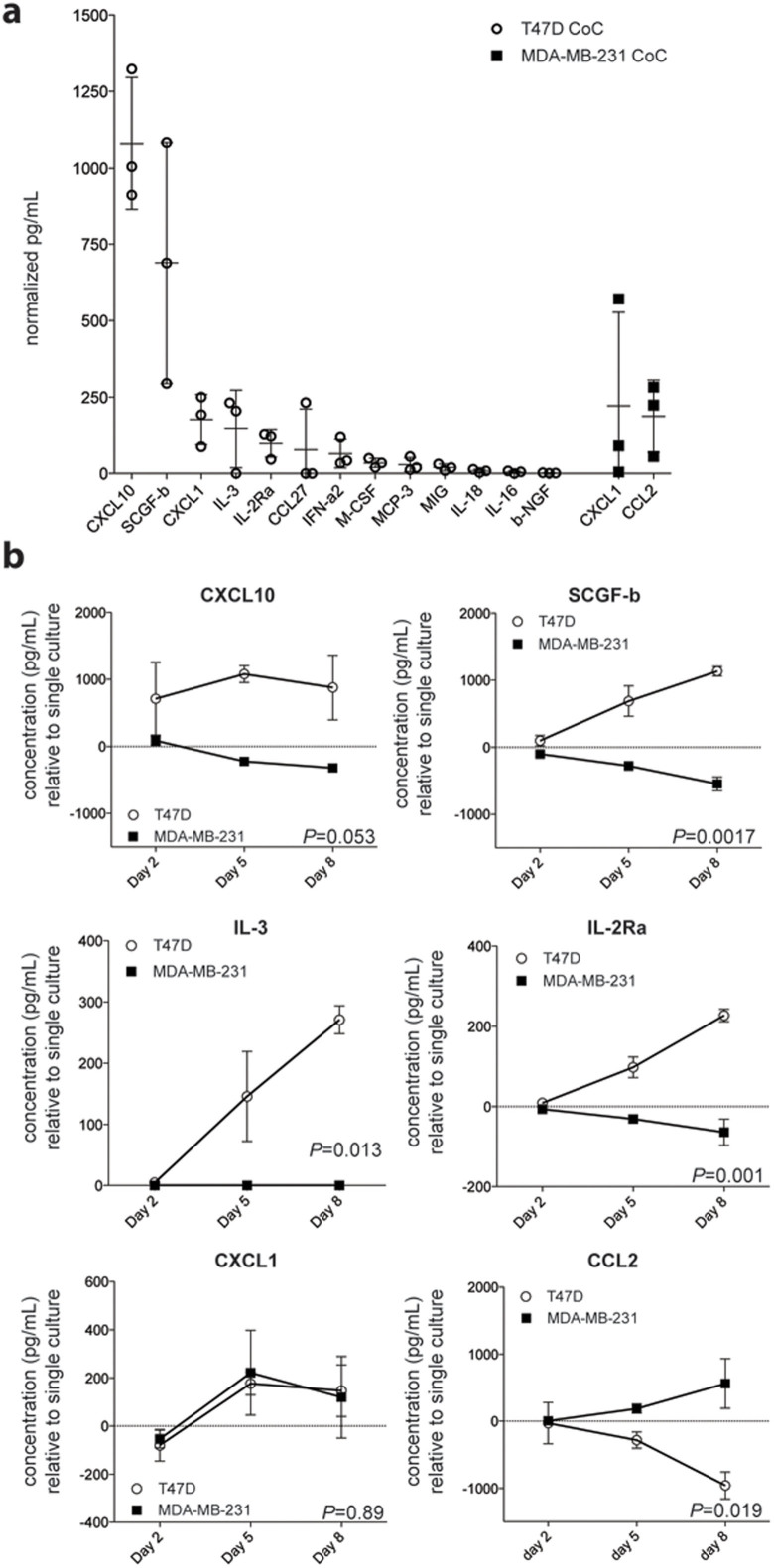
Secretion analysis of co-culture-induced factors. (a) Cytokine, chemokine and growth factor secretion analysis of co-culture conditioned medium (n = 3 donors) using Bio-plex pro assay Human Group I and Human Group II (BioRad). The concentration of the measured factors in the co-culture is shown as relative difference to the concentration of factors secreted from both single cultured cancer cells and monocytes. (b) Longitudinal measurements of secreted factors from co-culture medium comparing monocyte (n = 3) activation with T47D or MDA-MB-231 cells relative to individually cultured cells (two-way ANOVA).

To investigate how macrophage-cancer cell interactions in T47D and MDA-MB-231 cells might contribute to differential secretion of soluble mediators, we measured major cytokines, chemokines and growth factors in the co-culture conditioned media, using a panel of 48 factors included in Bio-plex pro assay Human Group I and Human Group II (BioRad). Because both cancer cells and monocytes secreted many factors of the panel when cultured alone, we measured the relative difference to obtain the relevant increase of a specific factor induced solely by the macrophage-cancer cell crosstalk (Fig. 5a, day 5 normalized mean from 3 donors). The factors induced by T47D co-culture (Coc) were mostly mediators of pro-inflammatory signals. The levels of CXCL10 (*P* = 0.053), IL-2RA (*P* = 0.001), IL-3 (*P* = 0.013) and stem cell growth factor (SCFG) -*β* (*P* = 0.0017) were increased during the co-culture period compared to MDA-MB-231 co-culture (Fig. 5b, n = 3, two-way ANOVA). Of note, IL-3 was the only factor that was not secreted by either cell type alone but induced only upon T47D co-culture. While CXCL1 secretion was activated in both co-cultures, a significant increase of CCL2 was only observed under MDA-MB-231 co-culture (Fig. 4b; *P* = 0.019, n = 3, two-way ANOVA).

### Macrophages suppress the proliferation of T47D but not MDA-MB-231 cells

**Figure 6:**
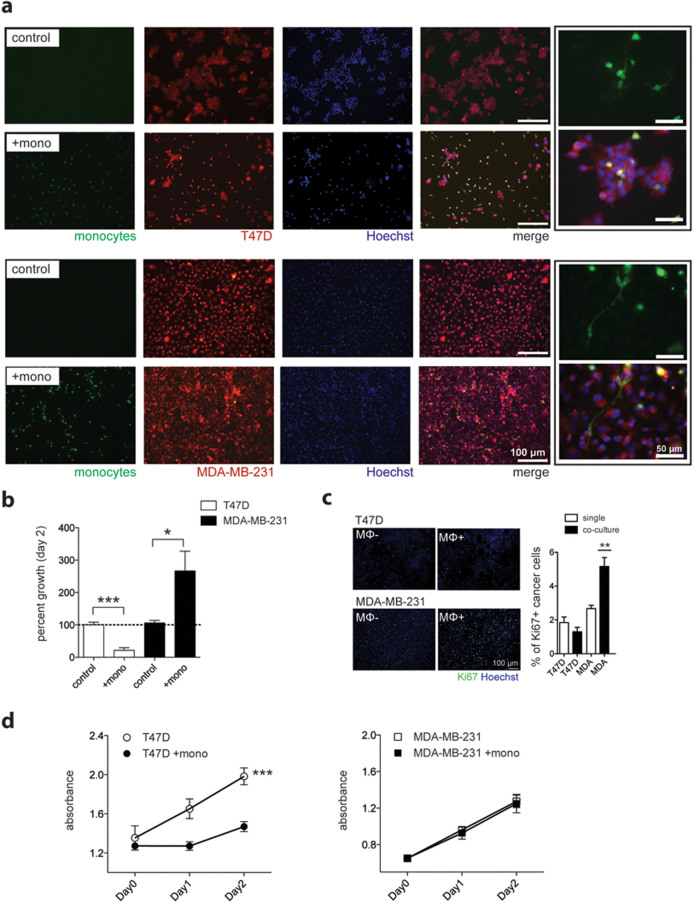
T47D-educated monocytes decrease breast cancer cell proliferation. (a) Cell tracker red labeled T47D and MDA-MB-231 cells were cultured in monolayers under starvation with (+mono) or without (control) the addition of freshly isolated cell tracker green labeled monocytes from three different donors. (b) The number of Hoechst positive nuclei from five microscopic fields (10×) per donor was counted and represented as mean value for each individual on day 2, normalized to the control wells. The monocyte Hoechst signal (Hoechst positive nucleus with green cytoplasma) was excluded from the analysis. (c) Ki67 staining of T47D and MDA-231 cells at day two, cultured under starvation with or without the presence of monocytes (MΦ). Cancer cell proliferation (Ki67 positive cells) was quantified from three independent experiments using three different donors. (d) MTT cell viability assay of unlabeled cancer cells cultured with or without monocytes (n = 3 donors, performed in triplicate wells) for indicated time points. *P≤0.05; **P≤0.01; ***P≤0.001.

Our results indicated that the T47D educated macrophages were more pro-inflammatory, suggesting that they also might have anti-tumor effects. To test this, we added freshly isolated monocytes on top of cancer cell monolayers and visualized cell confluence after two days. The cells were labeled with different colors (cancer cells, red; monocytes, green) to distinguish both populations. We observed a dramatic decrease in cell confluence of T47D cells after two days in co-culture compared to T47D cells cultured alone (Fig. 6a and b; *P* = 0.0003, Student's *t*-test). Most of the monocytes, originally labeled in green, became also visible in the red channel, indicating that they phagocytized T47D cell debris.

The MDA-MB-231 cells were influenced by the presence of monocytes in an opposite fashion and were more confluent than the single cultured cancer cells (Fig. 6 a and b; *P* = 0.01, two-tailed Student's *t*-test). This was reflected by a lighter red color, resulting from dimming of the fluorescence after each cell division. The monocytes/macrophages under direct MDA-MB-231 co-culture exhibited an elongated morphology and appeared mainly green (Fig. 6a, close up). Additionally, the number of Ki67^+^ MDA-MB-231 cells increased after addition of monocytes (MΦ) compared to MDA-MB-231 cells cultured alone (Figure 6c; n = 3 donors done as triplicates, *P* = 0.0062, two-tailed Student's *t*-test). No increase in Ki67 was observed in T47D cells. Using a different assay of cell proliferation and viability, we observed a significant suppression of T47D cell proliferation in the presence of monocytes compared to T47D cells cultured alone (Fig. 6d; *P* = 0.0003, two-way ANOVA), whereas MDA-MB-231 cells proliferated equally in both conditions (Fig. 6d).

## Discussion

Our observations demonstrate that different types of breast cancer cells per se have different abilities to activate macrophage functions independent of other stromal components. A few other reports have also described the importance of cancer-secreted factors, such as versican or cancer-secreted exosomes in stimulating metastasis and immunomodulation via Toll-like receptor 2 activation on myeloid cells and macrophages, respectively^25^,^26^. In our study, we found that the more aggressive MDA-MB-231 breast cancer cells promoted monocyte differentiation into M2-like macrophages, as represented by elongated cell morphology and up-regulation of the M2 marker MMR. These changes are similar to what has been reported for IL-4 activated macrophages^27^. In addition, whole transcriptome sequencing of MDA-MB-231 co-cultured macrophages in conjunction with pathway analysis indicated down-regulation of the citrulline metabolism pathway. L-citrulline is converted from L-arginine by inducible nitric oxide synthase (iNOS) after stimulation with pro-inflammatory cytokines (e.g. IL-1, tumor necrosis factor (TNF)–α and interferon gamma (IFN-γ)) to produce nitric oxide (NO). NO is an important mediator of angiogenesis and neovascularization^28^. It also activates immune responses against pathogens^29^, and increased expression of iNOS on macrophages represents a marker for M1 polarization^30^. Therefore, soluble factors secreted by MDA-MB-231 cells can efficiently suppress the activation of pro-inflammatory functions of human monocytes that otherwise would develop, for example under T47D co-culture, during the period of in vitro culture. In fact, monocyte activation by T47D cells resulted in a significantly reduced survival and proliferation of T47D cells but not of MDA-MB-231 cells. A similar selective human macrophage mediated cytotoxicity has been reported to develop within eight hours of co-culture after stimulating human monocyte-derived macrophages with IFN-γ^31^.

At the protein level, the secretion of CCL2 was increased in MDA-MB-231/macrophage co-culture. Many reports have proposed a tumor-promoting role for this chemokine, as it has been shown to mediate breast cancer metastasis^15^ and prostate cancer cell growth directly and indirectly via increased macrophage recruitment^32^. Most likely, cancer cell - macrophage crosstalk in TNBC initiates a vicious circle where both cell types reinforce each other^33^,^34^. This might also occur in ER^+^ tumors as they progress, as has been reported for colorectal cancer^35^. However, the initial response observed in T47D co-culture was more pro-inflammatory, as illustrated by the up-regulation of many pro-inflammatory genes, immune activation pathways and increased secretion of CXCL10, IL-2RA and IL-3. IL-3 is mostly secreted by activated T-cells and enhances the development of tumor-reactive cytotoxic cells by a CD4-dependent mechanism^36^. IL-3 can also activate macrophages to increase the expression of MHC class II molecules and IL-1^37^. The use of IL-3 has also been reported to enhance macrophage-dependent anti-tumor effects when utilized in combination with cancer gene therapy^38^.

The down-regulation of the thioredoxin pathway in T47D co-cultured macrophages suggests that these cells polarized to an M1-like phenotype since thioredoxin-1 has been shown to promote anti-inflammatory macrophages in a mouse model of atherosclerosis^19^. There was also a significant down-regulation in vitamin D signaling in both T47D cells and macrophages under co-culture conditions. Vitamin D_3_ has been shown to affect the immunomodulatory functions of human monocytes/macrophages^39^. In fact, vitamin D receptor (VDR) deficient mice show overproduction of IL-17 with more severe inflammatory bowel disease^40^. We found similarly an up-regulation of IL-17 signaling pathways in both T47D cells and T47D stimulated macrophages. These observations support our conclusion that T47D co-cultured macrophages have a pro-inflammatory phenotype and might indicate the presence of anti-tumor immune responses in ER^+^ cancers.

Because we focused our analysis exclusively on genes that were differentially regulated in macrophages activated by T47D versus MDA-MB-231 cells, it is relevant to note that at the RNA level, M2-polarization markers were expressed in both T47D and MDA-MB-231 activated macrophages, but were more strongly expressed in the latter. While tumor-conditioned macrophages have been reported to execute pro-tumor functions and express M2 markers, Grugan et al. reported that they still retain tumoricidal functions in the presence of tumor-targeting mAbs^41^, highlighting the plasticity of macrophage functions. One has to also keep in mind that the selected time point for sequencing, which was five days after co-culture, shows only a snapshot of the changes occurring in these cells over time, and many modifications that define M1/M2 polarization might have occurred earlier. There are currently no clinical studies directly investigating how the phenotype or the density of TAMs in ER^+^ and TNBC is differently associated with patient prognosis. However, in one report the density of CD163^+^ TAMs in the tumor stroma was positively correlated with triple-negative/basal-like breast cancer and inversely correlated with luminal A breast cancer^42^. The cancer cell induced differences in macrophage activation between T47D and MDA-MB-231 cells were likely due to the fact that these cell types represent opposite ends of breast cancer heterogeneity. It remains to be investigated how other TNBC subtypes mediate macrophage activation^43^ and if less aggressive breast cancer types also promote pro-inflammatory functions. Our findings suggest, however, that macrophage activation in breast cancer is not solely unfavorable and should be taken into consideration when using macrophage-targeting treatments.

## Methods

### Isolation of human peripheral monocytes

Peripheral blood mononuclear cells from healthy donors were obtained from a commercial source of blood products (Blutspende Zürich, Zurich, Switzerland), and therefore no experimental approval or informed consent was further needed. However, all samples were handled according to the regulations and guidelines of ETH Zürich. Monocytes were isolated from buffy coats by density gradient centrifugation using Ficoll-paque PLUS (GE Healthcare) and magnetically enriched by negative selection using Human Monocyte Isolation Kit II (MACS, Miltenyi Biotech). Monocyte purity > 95% was confirmed by FACS analysis with anti-CD14 staining (BD Biosciences).

### Transwell co-culture assay

Before experimentation, the breast cancer cell lines T47D and MDA-MB-231 (kindly provided by Dr. Nancy E. Hynes, FMI, Basel, Switzerland) were tested negative for mycoplasma with MycoProbe (R&D Systems). Cell line authentication was also confirmed by short tandem repeat (STR) profiling at Microsynth AG (Switzerland) to match the fingerprint of ATCC corresponding cell lines. 5 × 10^4^ or 1 × 10^6^ breast cancer cells/well were seeded in the lower compartment of 12 well transwell polyethylene terephthalate (PET) permeable supports or 75 mm polycarbonate transwell inserts pore size 0.4 *μ*m (Corning), respectively, and let to adhere o/n in DMEM supplemented with 10% FBS. The medium was replaced with RPMI (Gibco) one hour before adding 5 × 10^4^ (immunofluorescence) or 4 × 10^6^ (RNA-seq) freshly isolated human peripheral monocytes into the upper compartment of the transwell inserts. The co-cultures were incubated for indicated time periods without medium change in a humidified chamber at 37°C.

### RNA-Seq and gene expression

RNA from both macrophages (n = 3) and cancer cells (n = 3) in co-culture was isolated using Trizol (Invitrogen). The RNA samples were sent to BGI China and sequenced according to BGI standard protocols using Illumina HiSeqTM 2000. Donor 2 samples collected in single culture (2.52 μg) or with T47D cells (2.42 μg) did not reach a desired amount > 3 μg of RNA and were left out of the analysis. High-quality reads were aligned to the human reference genome with SOAPaligner/SOAP2^44^. The matched reads were then aligned to Human Refseq mRNA (NCBI). The expression levels for each gene were normalized to reads per kilobase per million mapped reads (RPKM) to facilitate the comparison of transcripts among samples. A mean log_2_Ratio (RPKM of macrophages (MΦ) co-cultured with MDA-MB-231 or T47D cells vs. MΦ single culture; RPKM of MDA-MB-231 or T47D cells co-cultured with MΦ/MDA-MB-231 or T47D cell line cultured alone of each gene was calculated. To identify genes differentially expressed between groups, an algorithm was used^45^ with a correction for false positive (type I) and false negative (type II) errors using the FDR method^46^. The genes were regarded as differentially expressed when their FDRs were less than 0.05. Further, genes were classified as up-regulated when their mean log_2_Ratio was larger than 0.5 or down-regulated when their log_2_ ratio was less than −0.5.

### Immunofluorescence staining

Human macrophages were stained with anti-CD68 (clone PG-M1, DakoCytomation) and anti-CD206 (MRC) (R&D Systems), both diluted 1:100 in Antibody Diluent (Zytomed Systems). The signal was detected using Alexa fluor 488 and 594 conjugated secondary antibodies (Molecular Probes), and the samples were imaged with an Axioskop 2 mot plus fluorescence microscope equipped with Plan-APOCHROMAT 20×/0.8 NA and 40×/0.95 NA objectives and an AxioCam MRc camera with AxioVision software 4.7.1 (Carl Zeiss AG).

### Multiplex assay

Conditioned media (serum-free) from the co-culture experiments (n = 3 donors) were analyzed for cytokine, chemokine and growth factor production using Bio-plex pro assay Human Group I and Human Group II (BioRad) according to the manufacturer's instructions. The co-culture cytokine levels are shown after subtraction of the added levels of individually cultured cancer cells and monocytes: *X_coc_ − (X_mono_ + X_cancer_) = X_relative change_*.

### Direct co-culture

T47D and MDA-MB-231 (5 × 10^4^) cells were labeled with CellTracker Red CMTP (Molecular Probes) according to the manufacturer's instructions and plated in 8 well culture slides (Corning) in DMEM supplemented with 10% fetal calf serum (FCS) the previous day. Two hours before adding CellTracker Green CMFDA (5-chloromethylfluorescein diacetate, Molecular Probes) labeled human monocytes at a ratio of 1:1 (initial amount of cancer cells:monocytes), the medium was replaced with RPMI without serum. The cells were cultured for two days, fixed with ice-cold methanol, stained with Hoechst and imaged for cell number changes. Five 10× microscopic fields for each donor (n = 3, in triplicates) were counted based on the Hoechst stain, using ImageJ software. Co-localization of green signal with Hoechst stain accounted for monocytes, which were therefore excluded from the analysis.

For Ki67 staining the cells in direct co-culture were left unlabeled and cultured as above. The cells were fixed with methanol and stained for Ki67 (clone B56 1:100, BD Biosciences) according to the above-mentioned protocol. To exclude that the added monocytes did not contribute to the Ki67 signal, the monocytes were cultured in parallel with cancer cell conditioned medium and stained for Ki67.

### Conditioned medium

Conditioned media were obtained from cultured cancer cells by plating 2 × 10^6^ cells in 10-cm cell culture plates in normal growth medium (DMEM, 10% serum) the previous day. The cells were let to adhere o/n and washed the next morning twice with PBS to remove the remaining serum, which was then replaced with RPMI (Gibco) only. The RPMI was incubated with or without (control) cells at 37°C for 24 hours. The conditioned medium was then collected and filtered through a 0.2 μm syringe and used directly.

### Cell proliferation assay

For CellTiter 96 Non-Radioactive Cell Proliferation Assay (MTT), 5 × 10^3^ T47D or MDA-231 cells were plated in 96-well plates in DMEM containing 10% FCS. The next day the cells were washed and RPMI was added with or without 5 × 10^3^ freshly isolated monocytes/well. Each treatment and time point was done in triplicates using monocytes from three different donors. The monocyte signal in single culture was very low (~0.08 absorbance) and did not change over time. Therefore, no normalization for the addition of monocytes was done. The wells were measured using a Sunrise plate reader (Tecan) at indicated time points according to the manufacturer's instructions (Promega).

### Statistical analyses

Results are presented as mean ± SD. Statistical significance between groups was determined as indicated in the text. The analyses were performed and the graphs were plotted in Prism V5.0 (GraphPad Software). A *P*-value of ≤0.05 was considered to be statistically significant.

## Author Contributions

M.H., F.R. and S.K. designed and performed experiments and analyzed data. M.H., F.R., S.K. and M.D. wrote the manuscript.
